# Feeding spray-dried plasma to broilers early in life improved their intestinal development, immunity and performance irrespective of mycotoxins in feed

**DOI:** 10.3389/fvets.2023.1321351

**Published:** 2024-01-11

**Authors:** Gabriela Gómez-Verduzco, José Arce-Menocal, Carlos López-Coello, Ernesto Avila-González, Claudia C. Márquez-Mota, Javier Polo, Luis Rangel

**Affiliations:** ^1^Departamento de Medicina y Zootecnia de Aves, Facultad de Medicina Veterinaria y Zootecnia, Universidad Nacional Autónoma de México, Ciudad de México, Mexico; ^2^Departamento de Producción avícola, Facultad de Medicina Veterinaria y Zootecnia, Universidad Michoacana de San Nicolás de Hidalgo, Morelia, Mexico; ^3^Centro de Enseñanza, Investigación y Extensión en Producción Avícola CEIEPAv, Tláhuac, Mexico; ^4^Departamento de Nutrición Animal y Bioquímica, Facultad de Medicina Veterinaria y Zootecnia, Universidad Nacional Autónoma de México, Ciudad de México, Mexico; ^5^APC LLC, Ankeny, IA, United States

**Keywords:** feed additive, intoxication, poultry, health, nutrition, growth, Immunity

## Abstract

**Introduction:**

Fungi that produce mycotoxins can grow on certain food products, such as grains and feed, and can cause a variety of health issues if consumed by animals, including chickens. The use of spray-dried plasma (SDP) is one strategy for combating the health problems caused by mycotoxins.

**Materials and methods:**

In the present study, Ross 308 chickens (*n* = 960) were divided into four treatment groups. T1 group was given a control diet (corn–soybean meal), T2 group was given a control diet +2% SDP, T3 group was given a control diet +2% SDP + mixture mycotoxins and T4 group was givena control diet + mycotoxin mixture.

**Results:**

The presence of SDP resulted in weight gain and decreased feed efficiency, whereas mycotoxins resulted in weight loss and increased feed efficiency. SDP increased the thymus’ relative weight. The presence of mycotoxins increased the heterophile/lymphocyte ratio. The presence of mycotoxins reduced the production of IL-2 and macrophage inflammatory protein-3 Alpha (MIP-3a), whereas the presence of SDP increased the production of macrophage colony-stimulating Factor (M-CSF). SDP resulted in higher IgA concentrations in the intestinal and tracheal washes than mycotoxin. Finally, adding SDP to broiler diets boosts weight gain, feed efficiency, and immune system development.

**Discussion:**

Our results provide information supporting that SDP is a promising tool for improving poultry immunity and performance.

## 1 Introduction

Grain consumption by the agricultural industry has increased significantly in recent years because of rising demand for high-quality animal protein to meet the needs of the world’s expanding population (1) and consistent increase is expected in the future. Grain storage and transport to various geographically distant regions have been linked to the development of mycotoxins that can be consumed by animals (2, 3).

The main ingredients used in poultry feed in intensive production systems are soybean meal mixed with several cereal grains, which are susceptible to mycotoxin contamination (4). Mycotoxin contamination in cereal grain is a major problem; it is estimated that more than 70% of cereal-based diets contain at least one mycotoxin (5). In most countries, the quantity of grains produced for animal feed is insufficient to satisfy their needs. As a result, grains are acquired from other regions or countries with long delivery periods, which favors the growth of fungi and the subsequent production of secondary metabolites such as mycotoxins (6–8). Mycotoxin poisoning in chickens has been associated with economic losses due to cross-border rejection in the local and global markets, which affects access to macro-and micronutrients, particularly in poor countries. Mycotoxins are produced by a variety of fungal species, including Aspergillus, Fusarium, and Penicillium. Among the well-known agricultural mycotoxins are aflatoxins (AF), fumonisins (FUM), zearalenone (ZEN), T-2 toxin (T-2), deoxynivalenol (DON), and ochratoxin A (OTA) (9, 10). Patulin, citrinin, sterigmatocystin, ergot alkaloids, and trichothecenes are harmful mycotoxins that can negatively impact animals, food, humans, and plants (11). These toxins pose a threat to animal health, leading to reduced productivity, impaired growth, reproductive problems, and, in severe cases, death. It is crucial to address the presence of these mycotoxins to safeguard the well-being of animals and ensure the safety of food and crops. Particularly in avian diets can negatively impact the immune system, gastrointestinal tract, liver, and other organs, resulting in decreased productivity and, in extreme situations, death (12). Furthermore, mycotoxin contamination in animal feed can result in the transfer of these toxins to animal products such as milk and meat, posing a risk to human health. Therefore, understanding the types of mycotoxins and their impact on animal health is crucial for ensuring food safety and maintaining the well-being of both animals and humans. Furthermore, the fact that mycotoxins are difficult to eliminate with thermal, chemical, or physical treatments is another cause for concern (4).

Therefore, the poultry industry needs feed additives that help to counteract the negative effects of mycotoxins in animal feed (13). Among these additives is spray-dried plasma (SDP), which is a complex mixture of functional proteins with antibacterial properties such as albumin, transferrin, immunoglobulins and glycoproteins, bioactive peptides, growth factors, amino acids, and other molecules of biological interest that, in addition to improving farm animal productivity, has positive effects on animal health and welfare (14, 15). The poultry industry has been characterized as a highly efficient production industry for converting protein of plant origin into protein of animal origin, and those in the industry constantly seek continuous improvement in all production processes. The biosafety protocols currently used in this industry represent an opportunity for improvement (5).

In murine models, rats treated with *Staphylococcus aureus* enteroxin B, an immune system activator, developed intestinal inflammation. (16). When rats were fed SDP (8%), they showed an improvement in intestinal health, which was characterized by stronger intestinal barrier integrity, which was associated with a decrease in proinflammatory cytokines such as interleukin 6 (IL-6) and tumor necrosis factor alpha (TNF-α) and an increase in anti-inflammatory cytokines such as interleukin 10 (IL-10) and transforming growth factor beta 1 (TGB-β1) (17). IL-10 is an anti-inflammatory cytokine that suppresses the activation and function of immune cells, as well as the expression of pro-inflammatory cytokines (18–20). In contrast, TGB-β1 regulates the magnitude and type of the immune response (21).

Recent studies have shown that the use of SDP in the feed of farm animals such as pigs (22, 23) and poultry (24) increases the total systemic antibody titers. Therefore, the use of SDP may be a suitable strategy to improve the humoral immune response induced by vaccines. Based on these reports, the purpose of this study was to examine the use of SDP in broilers that received a balanced feed with or without extra addition of mycotoxins during the first 7 days of the chick’s life and to evaluate their productive performance, intestinal health, and humoral and cellular immune responses.

## 2 Materials and methods

### 2.1 Facilities and care of experimental animals

The experiment was carried out in accordance with the guidelines of the Official Mexican Standard (NOM-033-SAG/ZOO-2014) for animal welfare, and the experimental protocols were approved by the Institutional Committee for the Care and Use of Animals of the School of Veterinary Medicine of the National Autonomous University of Mexico (CI-CUAE-FMVZ-UNAM MC-2017/1-14). The experiment was carried out on an experimental farm located in the state of Michoacán, Mexico, at a height of 1940 meters above sea level.

### 2.2 Experimental design and handling of animals

Nine hundred sixty mixed chicks (50% male, 50% female) of the Ross 308 lineage from the same hatchery and same breeder were used, all the chicks against Marek’s disease in the hatch. The chicks were kept in production until 42 days of age. The chicks were completely randomized into four experimental treatments, with 8 replicates and 30 birds per replicate. The T1 (−P, −M) group was fed a control diet (corn–soybean meal), the T2 (+P, −M) group was fed a control diet +2% SDP, the T3 (+P, +M) group was fed a control diet +2% SDP + mycotoxin mixture and the T4 (−P, +M) group was fed a control diet + mycotoxin mixture. The dosage of SDP was determined according to previous reports (25) and was supplemented in the diet. The mixture of mycotoxins added to the feed in treatment groups T3 and T4 was 2.5 parts per billion (ppb) aflatoxin, 4.14 ppb T-2, 0.66 ppb ochratoxin, 19.32 ppb zearalenone, 5.83 ppb fumonisin and 3.6 ppb deoxynivalenol (DON). The SDP was donated by APC proteins ®. Only the feed administered on the first 7 days (preliminary diet) was supplemented with mycotoxins. The feeding of the chicks was divided into four phases: starting: 1 to 7 days, growing: 8 to 21 days, finisher 1 22 to 35 days, and finisher 2 36 to 42 days, and they had *ad libitum* access to feed and water. Table 1 shows the formulation of the experimental treatments.

**Table 1:** Composition of the experimental diets.

Ingredients	Preliminary control	Preliminary plasma	Starter	Finisher 1	Finisher 2
	(0 – 7 days)	(0 – 7 days)	(7 – 21 days)	(21–35 days)	(35 – 42 days)
Yellow corn (7.5%)	604.7	634.1	630.5	650.6	674.8
Soy-bean meal (48%)	351.8	314.6	326.7	302.6	277.7
Spray-dried plasma (78%)	0.0	20.0	0.0	0.0	0.0
Limestone (40%)	11.4	11.7	10.9	10.1	9.3
Dical phos (21/17)	9.0	8.5	7.7	6.9	6.2
Pigment 30 g/kg	0.0	0.0	0.0	2.0	2.7
Soy oil	11.3	1.2	13.3	17.9	20.5
NaCl	3.7	2.6	3.8	3.8	3.8
DL-Methionine (99%)	2.9	2.7	2.6	2.0	1.4
Vit/Min premix**	3.0	3.0	3.0	3.0	3.0
Lysine HCl (78.6%)	1.2	0.8	1.0	0.5	0.1
Nicarbazine	0.5	0.5	0.5	0.0	0.0
Salinomycin	0.0	0.0	0.0	0.5	0.5
L-Threonine (99%)	0.4	0.1	0.0	0.0	0.0
Phytase Dupont	0.1	0.1	0.1	0.1	0.1
Total (kg)	1,000	1,000	1,000	1,000	1,000
Calculated composition					
Metabolizable energy(kcal/kg)	3,050	3,050	3,100	3,100	3,200
Crude protein (%)	22	22	21	20	19
Total calcium (%)	0.70	0.70	0.65	0.60	0.550
Available phosphorus (%)	0.45	0.45	0.42	0.4	0.380
Digestible Lys (%)	1.18	1.18	1.1	1.0	0.900
Digestible Met (%)	0.606	0.576	0.555	0.488	0.419
Digestible TSAA (%)	0.92	0.92	0.86	0.78	0.700
Digestible Trp (%)	0.227	0.236	0.214	0.202	0.189
Digestible Thr (%)	0.8	0.8	0.725	0.689	0.654
Digestible Ile (%)	0.959	0.939	0.911	0.863	0.815
Digestible Val (%)	0.995	1.023	0.949	0.902	0.856
Linolenic acid (%)	1.839	1.359	1.986	2.253	2.425
Total sodium (%)	0.2	0.2	0.2	0.2	0.200

Dical Phos: dicalcium/monocalcium phosphate, Vit/Min: Mineral and vitamin premix provided: vitamin A 12,000,000 IU, vitamin D3 2,500,000 IUP, vitamin E 15,000 IU, vitamin K3 2000 mg/kg, vitamin B1 2,250 mg/kg, vitamin B2 7,500 mg/kg, vitamin B3 45,000 mg/kg, vitamin B5 12,500 mg/kg, vitamin B6 3,500 mg/kg, vitamin B12 20 mg/kg, folic acid 1,500 mg/kg, biotin 125 mg/kg, iodine 300 mg/kg, selenium 200 mg/kg, cobalt 200 mg/kg, iron 50,000 mg/kg, copper 12,000 mg/kg, zinc 50,000 mg/kg, manganese 110,000 mg/kg. ** BHT(1.2%) and BHQ (9%).

To evaluate the systemic immune response, chickens were simultaneously vaccinated with a live virus vaccine against Newcastle disease through the intraocular route and an inactivated virus vaccine against Newcastle disease through the subcutaneous route (La Sota® Newcastle strain Laboratorios Avilab, SA and Newcastle Plus®, Laboratorios Avilab, S. A de CV, Tepatitlán de Morelos, Jalisco, Mexico.)

### 2.3 Determination of mycotoxins in treatments

The mycotoxin content was determined according to VICAM technology as previously reported (26). Table 2 shows the mycotoxin content in the treatments.

**Table 2:** Mycotoxin content in experimental treatments.

Treatments	Aflatoxin	Toxin T-2	Ochratoxin	Zearalenone	Vomitoxin	Fumonisin
	ppb					
T1 (−P, −M)	3.63	27.05	1.90	126.28	415	790
T2(+P, −M)	4.44	28.93	1.81	162.66	335	880
T3(+P, +M)	5.36	37.07	3.95	213.17	422	980
T4(−P, +M)	5.48	46.00	4.73	256.03	540	960
MTV	15.0	150.0	75.0	35,000	15,000	7,500

T1: control diet (corn–soybean meal), T2: control diet + 2% SDP, T3 control diet + 2% SDP + mycotoxin mixture, T4 control diet + mycotoxin mixture. MTV: maximum tolerable value Waters Corporation ™- VICAM; UK.

### 2.4 Productive performance

Chickens and feed were weighed weekly until Day 42 of age. Weight gain, feed consumption and feed conversion index were determined. Mortality was recorded daily.

### 2.5 Morphometric index

The morphometric index of the spleen, thymus, bursa of Fabricius and liver was determined on Days 21 and 42 by weighing each organ of 8 chickens from each treatment group with a scale (precision balance FPRS223, Thermo Fisher Scientific Inc., Germany.). The morphometric index was calculated as previously reported (27).

### 2.6 Evaluation of intestinal contact surface area

At 21 days of age, 8 birds were taken from each treatment group and the jejunum and ileum were isolated and analyzed. The data were obtained and processed according previously reported methods (28) with support from the Motic Images Plus 2.0 program (Routine Software Series, Motic Asia, Hong Kong).

### 2.7 Measurement of serum interleukin and chemokine levels

The serum levels of IL-2, M-CSF, and MIP-3α were determined at 14 days of age with a commercial ELISA test (Cat GCYT1-16 K. Millipore Corporation Merck; Darmstadt, Germany). Blood samples were taken from the left radial vein of the birds; 1 bird per replicate. The sera were handled as previously reported (29).

### 2.8 Measurement of total tracheal and intestinal IgA concentrations and serum IgY concentration

To quantify the total and nonspecific production of IgA in the epithelia of the trachea and jejunum, a commercial antigen capture ELISA chicken IgA quantification kit (Bethyl Laboratories, Inc., Montgomery, TX, USA) was used following the manufacturer’s recommendations. At 21 and 42 days of age, 8 broilers were sacrificed by anesthesia overdose. Ten-centimeter sections of jejunum and 3 cm of trachea were removed from each broiler. This process was performed according to what was previously reported (30). Serum samples were collected on day 14, 28 and 42 to measure IgY using the commercial available ELISA kit (Neo Biolab, Cambridge MS, Catalog AB157693) following the manufacturer’s recommendations.

### 2.9 Blood heterophil/lymphocyte index (H/L)

The H/L index in blood was determined on Days 25 and 40. Blood from 3 birds/treatment was collected from the radial vein of the wing and stored in EDTA-coated tubes, and then these samples were processed as previously reported (31).

### 2.10 Evaluation of the systemic humoral immune response

The humoral immune response was determined by the quantification of antibodies against Newcastle disease through the ELISA method at 14 days of age.

On Day 10 of age, the first immunization was carried out, and on Day 17 of age, a second immunization was carried out, only with the emulsified vaccine. Two milliliters of serum of eight chickens were taken from each treatment. Serum samples were collected and frozen at −20°C to determine the specific serum antibody titers against the Newcastle virus by the hemagglutination inhibition test (32).

### 2.11 Statistical analysis

Data were analyzed to verify the fulfillment of normality and homogeneity of variance assumptions (33). The results were analyzed using a completely randomized design, with a factorial arrangement of 2 × 2 treatments. One factor was the diet with and without 2% plasma (from 1 to 7 days of age), and the other factor was the diet with and without a mixture of mycotoxins. The comparison of means was performed by the Tukey test (StatSoft. Statistica version 10.0, 2011) and *p* < 0.05 was considered statistically significant.

## 3 Results

### 3.1 Body weight development, feed intake, feed conversion and mortality

As shown in Table 3, the addition of 2% SDP caused an increase in body weight on Day 7 (*p* < 0.021) and on Day 42 (*p* < 0.001), the T4 group had the lower (*p* < 0.001) body weight in comparison with T1, T2 and T3.Mycotoxins in the feed resulted in a decrease in body weight that could be observed from 7 days of age until 42 days of age (*p* < 0.05). As shown in Table 3, the inclusion of 2% SDP did not affect chicken feed intake in the period from 7 to 42 days, while the inclusion of mycotoxins caused an increase in feed intake (*p* < 0.032) at the end of the study (Day 42). At 7 days old, animal feed with T4 showed a higher feed conversion (*p* < 0.001) in comparison with T1, T2 and T3. Inclusion of 2% SDP causes a lower feed conversion on Days 7 and 42. Interestingly, feed conversion increased (*p* < 0.05) during the entire production cycle when mycotoxins were included in the food (Table 3). No differences in mortality were observed in the chickens in the present study (Table 3).

**Table 3:** Body weight development, feed intake, feed conversion at 7 and 42 days, and mortality at 42 days of age of male and female chickens fed 2% plasma and mycotoxins.

Factor		Body weight (Kg)		Feed intake (Kg)		Feed conversion		Mortality (%)
		7 days	42 days	7 days	42 days	7 days	42 days	42 days
Plasma (P)								
+ P		0.152^a^	3.058 ^a^	0.124	4.896	1.108^**b**^	1.622^**b**^	5.42
−P		0.145^b^	3.008^b^	0.129	4.89	1.227^**a**^	1.648^**a**^	6.04
Probability **		0.021	0.001	0.064	0.836	0.001	0.016	0.555
Mycotoxin (M)								
+ M		0.148	3.019^b^	0.128	4.925^a^	1.199^a^	1.653^**a**^	5.63
−M		0.150	3.046^a^	0.125	4.860^b^	1.136^**b**^	1.617^**b**^	5.83
Probability **		0.48	0.023	0.157	0.032	0.023	0.001	0.844
−P	− M	0.151^a^	3.025	0.125	4.841	1.125^**b**^	1.621	6.25
− P	+ M	0.140^b^	2.99	0.133	4.938	1.329^**a**^	1.674	5.83
+ P	− M	0.148 ^ab^	3.067	0.124	4.879	1.147^**b**^	1.612	5.42
+ P	+ M	0.156 ^a^	3.048	0.124	4.912	1.069^**b**^	1.633	5.42
Probability **		0.001	0.472	0.162	0.278	0.001	0.128	0.844
Mean		0.149	3.033	0.127	4.893	1.167	1.635	5.73
SEM***		0.002	0.007	0.001	0.015	0.021	0.006	0.501

P, SDP; M, mycotoxins, +, added, − without. Results of a 2 × 2 factorial arrangement; one factor was the diet with and without 2% plasma (from 1 to 7 d of age) and the other factor was the diet with and without mixed mycotoxins. When there were significant differences (5%), between the treatments, the comparison of means was made by Tukey’s test. Values with different sides are significantly different according to the probability indicated in the standard table. a>b.

Morphometric and H/L indices On Day 42, the addition of 2% SDP resulted in a 1.09-fold increase in the relative weight of the thymus when compared to no SDP inclusion (Table 4). There were no differences in the relative weights of the spleen, bursa, or liver with any of the interventions. The hemogram results revealed that the heterophil/lymphocyte index (H/L) was higher (*p* < 0.001) in the serum of birds given 2% SDP (T2) compared to birds fed mycotoxins (T3 and T4) or no mycotoxins (T1) at 25 days of age (Table 4). The presence of mycotoxins increased the H/L index at 40 days (*p* < 0.001).

**Table 4:** Relative weight expressed in (%) of the spleen, thymus, bursa of fabricio and liver with respect to live body weight at 42 days of age of male and female chickens fed with 2% plasma and mycotoxins.

FACTOR		Relative weight (%)				Heterophile/lymphocyte ratio	
		Spleen	Thymus	Bursa	Liver		
Plasma (P)		42 days				25 days	40 days
+ P		0.126	0.187^a^	0.115	1.733	2.39^a^	1.93
− P		0.124	0.171^b^	0.106	1.799	0.59^b^	1.88
Probability **		0.719	0.033	0.266	0.089	0.001	0.85
Mycotoxin (M)							
+ M		0.119	0.173	0.107	1.763	0.80^b^	2.43^a^
− M		0.132	0.185	0.114	1.769	2.18^a^	1.38^b^
Probability **		0.056	0.128	0.34	0.874	0.002	0.001
− P	− M	0.132	0.172	0.107	1.815	0.29^b^	1.29
− P	+ M	0.116	0.169	0.106	1.783	0.89^b^	2.47
+ P	− M	0.131	0.198	0.122	1.723	4.07^a^	1.47
+ P	+ M	0.121	0.177	0.108	1.743	0.70^b^	2.39
Probability **		0.693	0.223	0.375	0.505	0.001	0.652
Mean		0.125	0.179	0.111	1.766	1.49	1.91
SEM***		0.003	0.004	0.004	0.019	0.47	0.197

And heterophil/lymphocyte (H/L) ratio at day 25 and 40 of age. P, SDP; M: mycotoxins, +, added, − without. Results of a 2 × 2 factorial arrangement; one factor was the diet with and without 2% plasma (from 1 to 7 d of age) and the other factor was the diet with and without mixed mycotoxins. When there were significant differences (5%), between the treatments, the comparison of means was made by Tukey’s test. Values with different sides are significantly different according to the probability indicated in the standard table. a>b.

### 3.2 Evaluation of the intestinal contact area surface in the duodenum, jejunum, and ileum

As shown in Table 5, in chickens treated with 2% SDP, the surface capacity of the duodenum contact area increased 4.0% (*p* < 0.033) compared with that in chickens not treated with SDP. Interestingly, the surface capacity of the duodenal contact area decreased by 6% in chickens that were fed a diet with high mycotoxin levels (*p* < 0.004) compared to chickens that were fed a diet with low mycotoxin levels.

**Table 5:** Surface capacity contact area of duodenum, jejunum, and ileum from male and females chickens at 21 days of age fed 2% plasma and mycotoxins.

Factor		Surface capacity contact area (Micron^2^)		
		Duodenum	Jejunum	Ileum
Plasma (P)				
+ P		9.9^a^	6.8	6.9^a^
− P		9.5^b^	6.6	6.5^b^
Probability **		0.033	0.272	0.001
Mycotoxin (M)				
+ M		9.4^b^	6.4^b^	6.4^b^
− M		10.0^a^	7.0^a^	7.0^a^
Probability **		0.004	0.001	0.001
− P	− M	9.2	6.9^abc^	6.4^bcd^
− P	+ M	9.6	6.4^bc^	7.0^abc^
+ P	− M	9.9	6.9^abc^	7.5^a^
+ P	+ M	10.3	7.0^abc^	6.5^bcd^
Probability **		0.653	0.001	0.001
Mean		9.7	6.7	6.7
SEM**		0.106	0.073	0.067

P, SDP; M, mycotoxins, +, added, − without. Results of a 2 × 2 factorial arrangement; one factor was the diet with and without 2% plasma (from 1 to 7 d of age) and the other factor was the diet with and without mixed mycotoxins. When there were significant differences (5%), between the treatments, the comparison of means was made by Tukey’s test. Values with different sides are significantly different according to the probability indicated in the standard table. a>b>c.

As shown in Table 5, supplementation with 2% SDP did not alter the contact area of the jejunum, while the jejunum contact area of chickens that were fed a diet with mycotoxins decreased by 8.5% (*p* < 0.001) compared to that of chickens fed a diet without mycotoxins.

As observed in Table 5, supplementation with 2% SDP increased (*p* < 0.001) the surface capacity of the ileum by 5.9% compared to non-supplemented diets, while diets with mycotoxins decreased the surface capacity by 13.5% (*p* < 0.001) compared to diets that did not have mycotoxins.

### 3.3 Serum interleukin and chemokine levels

Table 6 shows that there are no differences in IL-2 and MIP-3 levels between chickens that received mycotoxins in their feed and those that did not. M-CSF concentration was increased in chickens treated with 2% SDP (*p* < 0.001).

**Table 6:** Evaluation of the immune response, through the measurement of IL-2, MIP-3α and M-CSF from male and female chickens 14 days of age fed with 2% plasma and mycotoxins.

Factor		IL-2	MIP-3α	M-CSF
		(pg/mL)		
Plasma (P)				
+ P		4344.1	237	703.0^a^
− P		4056.8	236.7	624.8^b^
Probability **		0.403	0.997	0.005
Mycotoxin (M)				
+ M		3754.0^b^	126.8^b^	666.1
− M		4646.9^a^	346.9^a^	661.7
Probability **		0.013	0.013	0.867
− P	- M	4371.3	347	563.9^c^
− P	+ M	3742.3	126.4	685.8^ab^
+ P	- M	4922.5	346.8	759.6^a^
+ P	+ M	3765.7	127.1	646.5^bc^
Probability **		0.441	0.996	0.001
Mean		4200.4	236.8	663.9
SEM***		183.1	44.38	17.67

P, SDP; M, mycotoxins, +, added, − without. Results of a 2 × 2 factorial arrangement; one factor was the diet with and without 2% plasma (from 1 to 7 d of age) and the other factor was the diet with and without mixed mycotoxins. When there were significant differences (5%), between the treatments, the comparison of means was made by Tukey’s test. Values with different sides are significantly different according to the probability indicated in the standard table. a>b>c.

### 3.4 Assessment of the systemic humoral immune response

**Figure 1:**
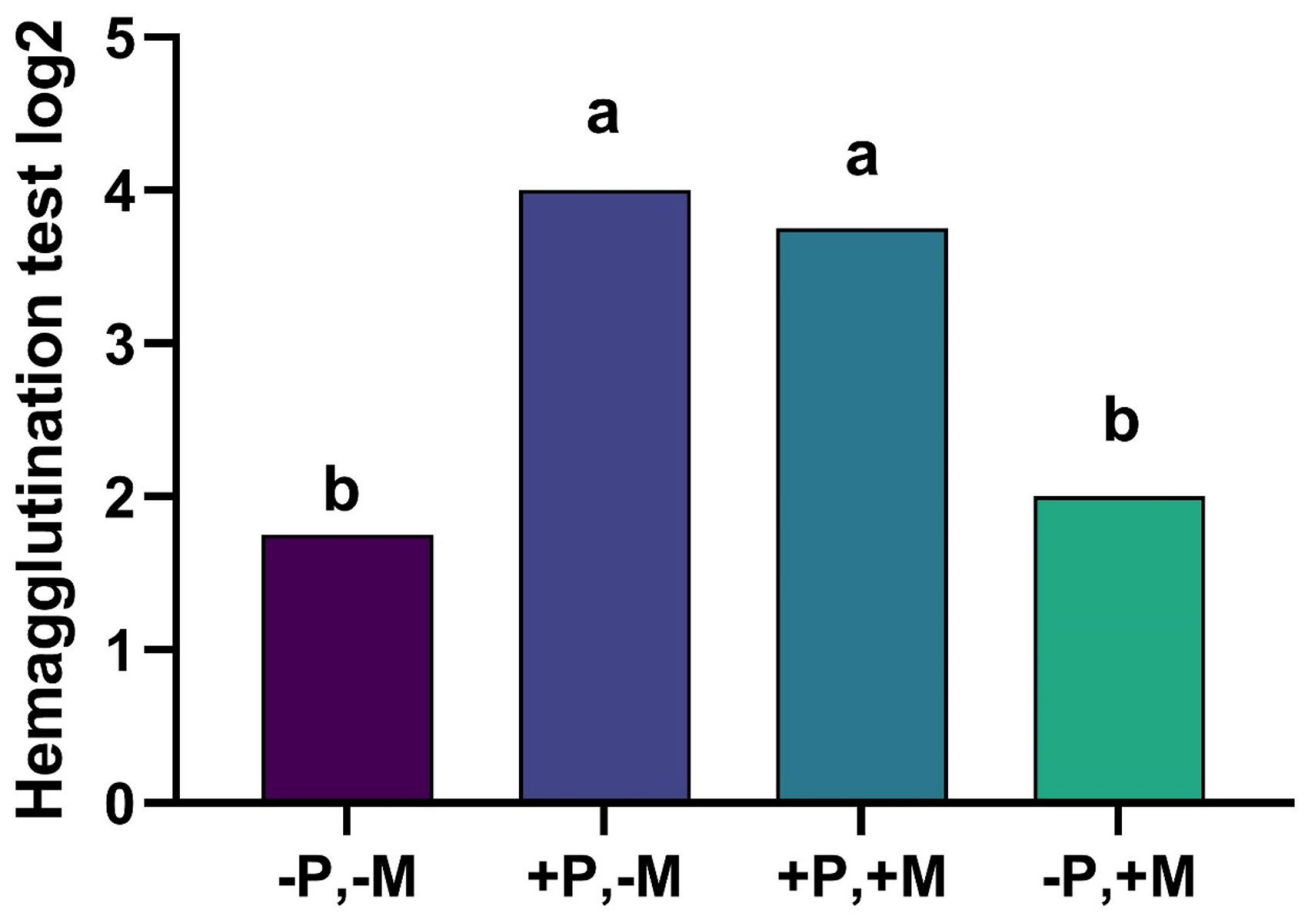
Assessment of the hemagglutination inhibition from male and female chickens 14 days of age fed with 2% plasma and mycotoxins. (−P without SDP, +P with SDP, −M without mycotoxins, +M with mycotoxins). Values are means. Labeled means without a common letter differ, *p* < 0.05, a > b.

The results of the hemagglutination inhibition test on the 14th day of life show that T2 and T3 increased total serum antibody response by 2.0 and 1.8-fold, respectively, when compared to T4 (Figure 1).

### 3.5 Measurement of total tracheal and intestinal IgA and serum IgY concentration

**Figure 2:**
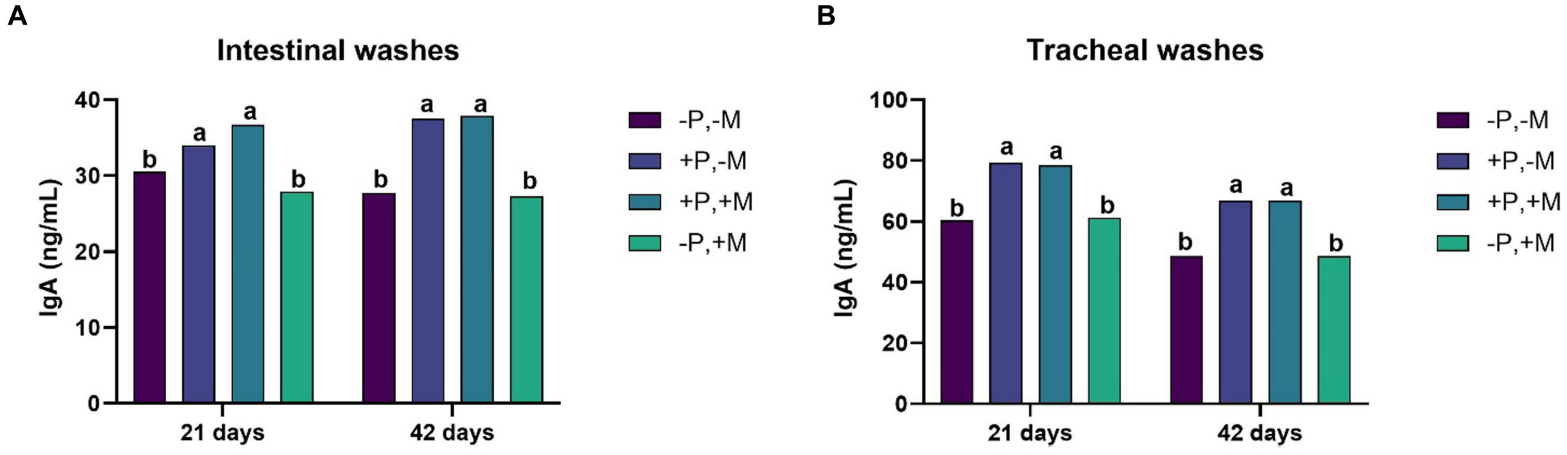
Assessment of the IgA concentration from intestinal washes **(A)** and tracheal washes **(B)** from male and female chickens of 21 and 42 days of age fed with 2% plasma and mycotoxins. (−P without SDP, +P with SDP, −M without mycotoxins, +M with mycotoxins). Values are means. Labeled means without a common letter differ, *p* < 0.05, a > b.

**Figure 3:**
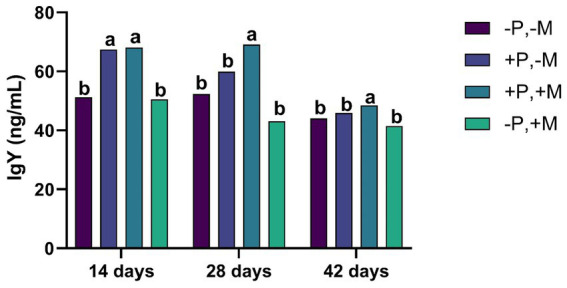
Assessment of the IgY from male and female chickens of 14, 28 and 42 days of age fed with 2% plasma and mycotoxins. (−P without SDP, +P with SDP, −M without mycotoxins, +M with mycotoxins). Values are means. Labeled means without a common letter differ, *p <* 0.05, a > b.

On Days 21 and 42, it was observed that the inclusion of 2% SDP (T2 and T3) significantly increased (*p* < 0.001) the concentration of IgA in intestinal and tracheal washes. Interestingly, in this same period, the inclusion of mycotoxins (T4) decreased (*p* < 0.01) the IgA titers both in the intestine and in the trachea (Figures 2A,B). The inclusion of mycotoxin (T4) caused a decrease (*p* < 0.001) in the concentration of serum IgY on Days 14, 28 and 42 (Figure 3).

## 4 Discussion

The first diet provided to chicks is critical for their growth and development, and this primary diet also contributes to the establishment of their microbiota (3) and plays an important role in the development of their immune response (34). Our results showed that feeding with SDP during the first 7 days of life promotes weight gain and improves the feed conversion rate, even when there are mycotoxins in the diet, which is of great importance given that the rate of feed conversion indicates the efficiency by which animal bodies use food to achieve optimal weight gain (35). The benefit of plasma in the primary diet was reflected in a greater weight of the chickens and a lower rate of conversion at sacrifice.

Mycotoxins are the secondary fungal metabolites of certain toxigenic fungal species which are proved to be detrimental for the health conditions of humans, animals, and birds. To date, approximately 400 different chemically diverse mycotoxins have been identified, but ochratoxins and aflatoxins are thought to be the most lethal in the poultry industry (36, 37) and these two mutagens have been reported as major carcinogens by International Agency for Research on cancer (38). The presence of mycotoxins in poultry diets has been associated with an increase in mortality rates, as well as a decrease in productive efficiency (39). Our results did not show a clear effect of mycotoxin on mortality during the first week of the study, so it is possible that a higher concentration is required to observe these differences. It has been reported that administering plasma in the pre started diet of chicks had no effect on carcass performance under normal conditions; however, when the chicks were raised in a stressful situation created by using the litter from the previous experiment, the presence of SDP in the diet increased meat yield (40). Therefore, the findings of this study could imply that mycotoxin contamination in the diet did not create a stressful situation sufficient to observe an effect of SDP addition on meat quality.

However, chickens fed mycotoxins had a higher consumption at 42 days, which resulted in a higher conversion rate at 42 days. (*p* < 0.001), demonstrating that the presence of mycotoxins, as previously stated, the mycotoxins are capable of impair animal productivity by affecting weight gain and reducing feed conversion.

The results obtained in the present study indicate that the use of 2% SDP in the first 7 days of life may be a promising strategy to ensure the development of broilers regardless of the presence of mycotoxins, provided that the mycotoxin level in cereal-based feed does not exceed the maximum level that can be biologically tolerated by birds. Similar results have been obtained in pigs (23, 41) in which piglets diets where contaminated with different levels of mycotoxins and observed that the presence of SDP mitigated the negative effects of the added mycotoxins on the growth of the animals.

In birds, the consumption of feed, as well as the absorption of nutrients, is highly regulated by the conditions of the gastrointestinal tract (42). Moreover, the prime responsibility of intestinal mucosa is the digestion and/or absorption of nutrients ultimately and a healthy mucosa ensures the optimum growth of the organisms (43). The inclusion of plasma improved the surface capacity of the contact area in the duodenum and ileum at 21 days meanwhile the presence of mycotoxins caused a detrimental effect in the surface capacity of the contact area in the duodenum, jejunum, and ileum. The contact surface capacity develops according to the growth of the chicks, and in our study, it is interesting to note that the absorption capacity of the distal segments (jejunum and ileum) increased with time and with the addition of SDP, which indicates that the use of plasma in the first days of life may be a promising strategy to improve intestinal function; however, more studies are needed to test this hypothesis. In general, the improvement in intestinal absorption capacity due to the presence of SDP positively correlated with length of the microvilli, especially at the ileum level. A study (44) in pigs demonstrated that the presence of SDP increased the surface area of intestinal microvilli due to an increase in their length instead of an increase in crypts. The greater presence of mycotoxins in the diet during the first 7 days was correlated with a lower absorption surface capacity in the duodenum, jejunum, and ileum, especially at 21 days, which could explain the lower growth observed in these birds during the study. The observed increase in the concentration of IgY can be associated with the greater relative weight (%) of the thymus as previously reported (45).

As previously mentioned, the presence of mycotoxins in grains destined for animal consumption can have detrimental effects on health due to the complex immune response that they provoke to these compounds (46). In the present study, we evaluated the response of different cytokines to the presence of mycotoxins and plasma. The presence of mycotoxins in the diet decreased (*p* < 0.004) the concentration of IgY, which is the main isotype that occurs in chickens (47), from 14 days of age, while the animals fed SDP presented a higher (*p* < 0.001) concentration of this immunoglobulin at 14 days of age. The observed increase in the concentration of IgY can be associated with the greater relative weight (%) of the thymus in the birds that were consuming SDP in the preliminary feed the first 7 days of life, which would probably also explain the higher titer of antibodies reported by the hemagglutination inhibition method at 14 days of age against Newcastle disease virus in birds that received SDP in diets with and without mycotoxins. This finding is interesting due to a possible stimulating effect on this primary lymphoid organ, and with it some immunostimulatory effects on the release of interleukins and chemokines that regulate and modulate the immune response (48). In chicks challenged with *Salmonella sofia* that the presence of plasma had no effect on the differences in the weights of the organs related to the immune system such as the liver, spleen and thymus, while the weight of the bursa of Fabricius was increased (25). In our study, an increase due to supplementation with SDP was observed in the thymus (*p* < 0.033), although numerically, the bursa of Fabricius also increased.

Regarding interleukins and cytokines, lower concentrations of IL-2 and MIP-3α were found in the serum at 14 days of age in the animals that consumed the diet with mycotoxin supplementation. These results are interesting since IL-2 is described as a growth factor of T lymphocytes, which induces all types of lymphocyte subpopulations and activates the proliferation of B lymphocytes (49) and therefore its effective action. In the literature, MIP-3α is described to be produced in the mucosa and skin by activated epithelial cells that attract activated B cells and memory T cells (50). Therefore, this decrease in birds that consume mycotoxins could be interpreted as an immunosuppressive effect at the immune level (51).

In contrast, chickens that consumed plasma had increased M-CSF levels, which is considered a macrophage colony-stimulating factor secreted by hematopoietic stem cells to differentiate into macrophages or other related cell types (52). In general, the observed interleukin and cytokine results indicate the potential of plasma to modulate the immune response and the importance of supplying it in the first days of life.

It is interesting to observe that at 25 days of age, the supplementation of SDP was associated with an increase in the H/L indices that could indicate an increase in innate immunity, while the presence of mycotoxins reduced it. It was observed in pigs fed diets contaminated with mycotoxins that the presence of these mycotoxins reduced the number of leukocytes and monocytes (53), as our results seem to indicate.

The results of the IgA concentration analysis in the intestinal and tracheal washes at 21 and 42 days of age indicate that the inclusion of SDP in the preliminary diet increased the titer of this immunoglobulin isotype, just the opposite effect to that observed with the inclusion of mycotoxins in feed that decreased IgA titers in both intestine and trachea. This information is important given that IgA is the isotype of immunoglobulin found in the mucosa, protecting at this level against antigens and toxins (54), and suggests that chicks receiving SDP in the diet have a greater immune capacity, as well as probably a stronger integrity of the gut barrier, as observed when chicks are subjected to systemic inflammation (55).

The current study adds to our understanding of the advantages of SDP in the feeding of various animal species. It is important to take notice that mycotoxin feed contamination is highly variable and therefore to gain more knowledge about the advantages of SDP we recommend the use of different levels of mycotoxin and different mixture of them. Further research, particularly in poultry, is required to assess its effect on intestinal health in poultry and its relationship with other types of diets.

## 5 Conclusion

The presence of mycotoxins in feed during the life cycle had negative effect on the growth of chicks probably due to poor intestinal development at this stage of their life and an alteration of the immune capacity that persists over time.

The inclusion of 2% SDP in preliminary corn-soy diets in broilers improves performance, intestinal health and immune response and reduces the negative effects associated with the presence of mycotoxins in the feed. The mitigating effects of SDP on mycotoxins are probably associated with the known effect of SDP to support and maintain a more efficient response of the immune system to inflammatory stress.

## Data availability statement

The raw data supporting the conclusions of this article will be made available by the corresponding author, upon reasonable request.

## Ethics statement

The animal study was approved by Institutional Committee for the Care and Use of Animals of the School of Veterinary Med-icine of the National Autonomous University of Mexico (CI-CUAE-FMVZ-UNAM MC-2017/1-14). The study was conducted in accordance with the local legislation and institutional requirements.

## Author contributions

GG-V: Methodology, Writing – original draft. JA-M: Conceptualization, Funding acquisition, Investigation, Project administration, Writing – original draft. CL-C: Methodology, Supervision, Validation, Writing – original draft. EA-G: Investigation, Methodology, Supervision, Visualization, Writing – original draft. CM-M: Writing – review & editing. JP: Conceptualization, Funding acquisition, Investigation, Project administration, Validation, Writing – review & editing. LR: Supervision, Validation, Writing – review & editing.
